# SMALL INTESTINAL L CELL DENSITY IN PATIENTS WITH SEVERE OBESITY AFTER ROUX-EN-Y GASTRIC BYPASS

**DOI:** 10.1590/0102-672020220002e1681

**Published:** 2022-10-03

**Authors:** Priscila Costa Estabile, Marco Aurélio Santo, Eduardo Guimarães Horneaux de Moura, Rogério Kuga, Priscila Caproni, Roberto de Cleva, Filippe Camarotto Mota, Fábio Quirillo Milléo, Roberto Ferreira Artoni

**Affiliations:** 1 Universidade de São Paulo, Postgraduate Program in Science in Gastroenterology – São Paulo (SP), Brazil; 2 Universidade de São Paulo, Faculty of Medicine, Hospital das Clínicas – São Paulo (SP), Brazil; 3 Universidade de São Paulo, Hospital das Clínicas, Faculty of Medicine, Gastrointestinal Endoscopy Service – São Paulo (SP), Brazil; 4 Universidade Estadual de Ponta Grossa, Department of Structural, Molecular and Genetic Biology – Ponta Grossa (PR), Brazil.

**Keywords:** Gastric Bypass, Immunohistochemistry, L Cell, Glucagon-Like Peptide 1, Diabetes Mellitus, Type 2, Derivação Gástrica, Imuno-Histoquímica, Células L, Peptídeo 1 Semelhante ao Glucagon, Diabetes Mellitus Tipo 2

## Abstract

**BACKGROUND:**

Enteroendocrine L cells can be found in the entire gastrointestinal tract and their incretins act on glycemic control and metabolic homeostasis. Patients with severe obesity and type 2 diabetes mellitus may have lower density of L cells in the proximal intestine.

**AIMS:**

This study aimed to analyze the density of L cells in the segments of the small intestine in the late postoperative of Roux-en-Y gastric bypass in diabetic patients with standardization of 60 cm in both loops, alimentary and biliopancreatic.

**METHODS:**

Immunohistochemistry analysis assays were made from intestinal biopsies in three segments: gastrointestinal anastomosis (GIA= Point A), enteroenteral anastomosis (EEA= Point B= 60 cm distal to the GIA) and 60 cm distal to the enteroenteral anastomosis (Point C).

**RESULTS:**

A higher density of L cells immunostaining the glucagon-1 peptide was observed in the distal portion (Point C) when compared to the more proximal portions (Points A and B).

**CONCLUSIONS:**

The concentration of L cells is higher 60 cm distal to enteroenteral anastomosis when comparing to proximal segments and may explain the difference in intestinal lumen sensitization and enterohormonal response after Roux-en-Y gastric bypass.

## RESUMO

**RACIONAL:**

As células L enteroendócrinas podem ser encontradas na extensão de todo trato gastrointestinal e suas incretinas atuam no controle glicêmico e da homeostase metabólica. Estudos mostram que pacientes obesos graves com diabetes mellitus tipo 2 apresentam má sinalização entero-hormonal e baixa resposta da secreção do peptídeo glucagon-1, que poderia ser explicado por uma densidade menor de células L ou uma distribuição mais distal ao longo do intestino delgado.

**OBJETIVOS:**

Analisar a diferença da densidade de células L nos segmentos do intestino delgado de pacientes obesos graves submetidos à gastroplastia em Y de Roux, em período pós-operatório tardio, com padronização de alça alimentar e biliopancreática com extensão de 60 cm em ambas.

**MÉTODOS:**

Ensaios de análises de imuno-histoquímica foram feitos a partir de biopsias intestinais obtidas em três segmentos: junto à anastomose gastrointestinal (AGI= Ponto A), junto à anastomose entero-enteral (AEE= Ponto B= 60 cm distal à AGI) e 60 cm distalmente à anastomose entero-enteral (Ponto C). Os resultados foram obtidos por meio de imunomarcação do peptídeo glucagon-1 secretado pelas células L.

**RESULTADOS:**

Foi observada maior densidade de células L na porção mais distal do intestino delgado (Ponto C) quando comparada às porções mais proximais (Ponto A e B).

**CONCLUSÕES:**

Em pacientes no pós-operatório de gastroplastia em Y de Roux, identificou-se concentração maior de células L já na porção a 60 cm distalmente a entero-entero anastomose quando comparada aos segmentos proximais, o que pode explicar diferenças na sensibilização no lúmen intestinal e na resposta entero-hormonal.

## INTRODUCTION

The enteroendocrine L cell produces the hormone glucagon-like peptide1 (GLP-1), with a prominent action on glycemic homeostasis and satiety control. It is found along the gastrointestinal tract (GIT), and its distribution varies according to the intestine segment. The density and location of the L cell may be of relevance for better understanding of the metabolic profile and diabetes mellitus control^4,8^.

These cells present in the mucosa are activated by a complex of internal and external stimuli. A poor signaling or low stimulation in enteroendocrine cells can directly affect metabolic activity and trigger the emergence of diseases such as obesity, metabolic syndrome, and type 2 diabetes mellitus (T2DM)^2^.

Previous studies in animal models (rats, pigs, cats, and dogs) showed incretin cells distribution along intestinal segments using polyclonal antibodies to perform qualitative or semi-quantitative assessment of the distribution of immunoreactive cells^16,17,18,19^. However, very few studies using human tissue from a small number of surgical biopsy specimens collected during Roux-en-Y gastric bypass (RYGB) confirmed the experimental results.

A study of L cell density and location made in cadavers demonstrated an increment in L cell density in distal portions of the jejunum and ileum in comparison with duodenum and proximal jejunum, and also an increasing density in proximal colon in comparison with the rectum^5^.

The distribution of L cells may also vary in healthy individuals and patients with T2DM. Immunostaining positive L cells in T2DM patients were more intense in the distal portion of the intestine and colon, while in normal individuals they are present in the proximal intestine and throughout the GIT^8^.

The treatment of patients with severe obesity by RYGB determines significant weight loss and significant improvement of glycemic metabolism due to an increased incretin release. Rhee et al. showed that anatomical changes lead to transcriptional modulation by altering the secretion of active enteroendocrine L cells after RYGB^18^.

There were no previous studies of L cell density and location in T2DM patients after RYGB. The objective of this study was to investigate the density of L cell in T2DM severely obese patients after Roux-en-Y gastroplasty, with standardization of the alimentary loop and biliopancreatic (60 cm in both).

## METHODS

A total of 14 patients with severe obesity (BMI≥40 kg/m^2^) and T2DM were prospectively evaluated after bariatric surgery at Metabolic and Bariatric Unit Hospital das Clínicas, Faculty of Medicine, Universidade de São Paulo.

This study was performed according to the ethical recommendations of the Declaration of Helsinki and was approved by the Human Research Ethics Committee of the Hospital das Clínicas, Faculty of Medicine, Universidade de São Paulo — Brazil, under process no. 324.454 (register 10799/2013).

### Acquisition of sample

Enteroscopic biopsies of the intestinal mucosa were performed in the late postoperative period of Roux-en-Y gastroplasty with standardization of the alimentary and biliopancreatic loops (60 cm in both). Intestinal biopsies were obtained in three segments: close to the gastrointestinal anastomosis (GIA= Point A), close to the enteroenteral anastomosis (EEA= Point B= 60 cm distal to the GIA), and 60 cm distal to the EEA (Point C).

### Immunohistochemical analysis

The tissue to be analyzed was embedded in paraffin and serial histological sections measuring 5 μm in thickness were cut on manual rotary microtome (Leica RM2125RT). The sections were dehydrated in an alcohol series, stained with hematoxylin and eosin, and mounted on slides.

To determine the immunohistochemical reactions, the histological sections were fixed on silanized slides (3-aminopropyl-triethoxysilane, Sigma R) and placed in an oven at 56°C for 24 h. The sections were then cleared in xylene (twice) at room temperature for 10 min per process and hydrated in decreasing concentrations of ethanol (100, 90, 70, and 50%), followed by a distilled water bath. Specific antigen recovery was performed for each antibody used. Table 1 lists the antibodies and respective technical details.

**Table 1:** Mean and median of L cells at points A, B, and C.

Point	Mean	Median (IQR)
A	5.4±3.0	6 (3–7)
B	6.6±5.3	5.5 (3–11)
C	12.6±4.73	11.5 (8–17)

IQR: interquartile range.

Antigen retrieval was performed in a microwave oven at full power for 20 min. A citrate buffer solution (10 mM citric acid, pH 6.0) was used for antigen recovery. The samples were left at room temperature to cool for 20 min. The histological cuts were then washed in running water for 5 min and incubated in 20 volumes of aqueous hydrogen peroxide solution changed once every 5 min (total of six times) to block endogenous peroxidase. A further 5-min washing in running water was performed and the sections were then washed three times (2 min per wash) with phosphate buffer saline (PBS).

The histological sections were incubated with the primary antibodies (previously diluted in PBS) for approximately 18 h (overnight) at 4°C. The dilution of the primary antibodies was 1:1000.

The sections were then washed with PBS three times (3 min per wash) and the reaction was revealed using the Novolink Polymer Detection System (Novocastra, UK). Incubation was performed with Post Primary Block for 30 min. The sections were washed in TBS for 2x5 min, followed by incubation with Novolink Polymer for 30 min and washing in TBS for 2x5 min, with gentle rocking. Peroxidase activity was developed with DAB working solution for 5 min. The slides were rinsed with water and the sections were counterstained with Carazzi hematoxylin, cleared in xylene, and mounted with Canada balsam.

For the negative control, slides containing histological sections underwent all steps of the immunohistochemical reaction, except incubation with the primary antibodies. The histological sections were analyzed using bright field microscopy (Olympus BX41) with a digital image capture system (Olympus DP71 equipped with DP-Controller software program). The images were treated using the Image Pro Plus 6.0 program. For validation purposes, immune cells were counted at 200x magnification with a random field for each slide for each antibody tested from all patients. Absolute cell numbers were compared by the paired t-test with the significance level set to 5% (p<0.05) using the GraphPad Prism 5.0 program^14^.

### Statistical analysis

The nonparametric Kruskal-Wallis test of independent samples with multiple comparisons adjusted by the Bonferroni method was used to analyze the L cell density by biopsy point. In multiple comparisons, the significance level of 1% (p=0.001) was adopted, with the L cell density being represented by mean+SD and median (IQR)^14^.

## RESULTS

**Figure 1:**
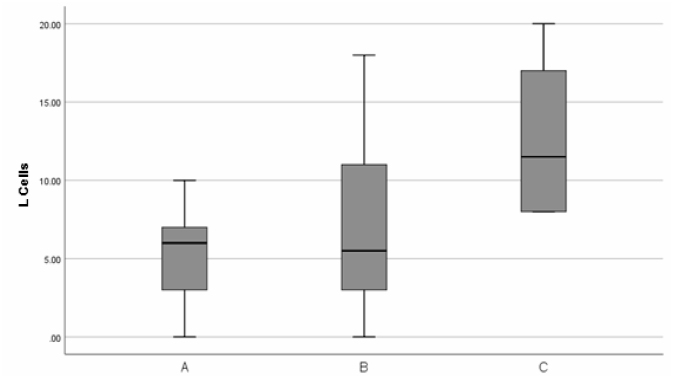
Distribution of cell count segments A, B, and C.

All intestinal biopsies showed normal histology. The mean density of L cells was as follows: point A= 5.4±3.0; point B= 6.6±5.3; and point C= 12.6±4.7. The same difference in the median (IQR) between points A, B, and C can be observed (Table 1 and Figure 1).

Multiple comparisons between the segments were performed without significant difference between points A and B (p=0.900). There was a significant difference between points A and C (p=0.001) and between points B and C (p=0.008).

It was observed that from the segment A at point B, active L cells are visible through positive immunostaining, while this cell density in the segment at point C is higher and more intense than in relation to point A, demonstrating a difference in active L cells which tends to increase at the most distal point (Figure 1).

**Figure 2:**
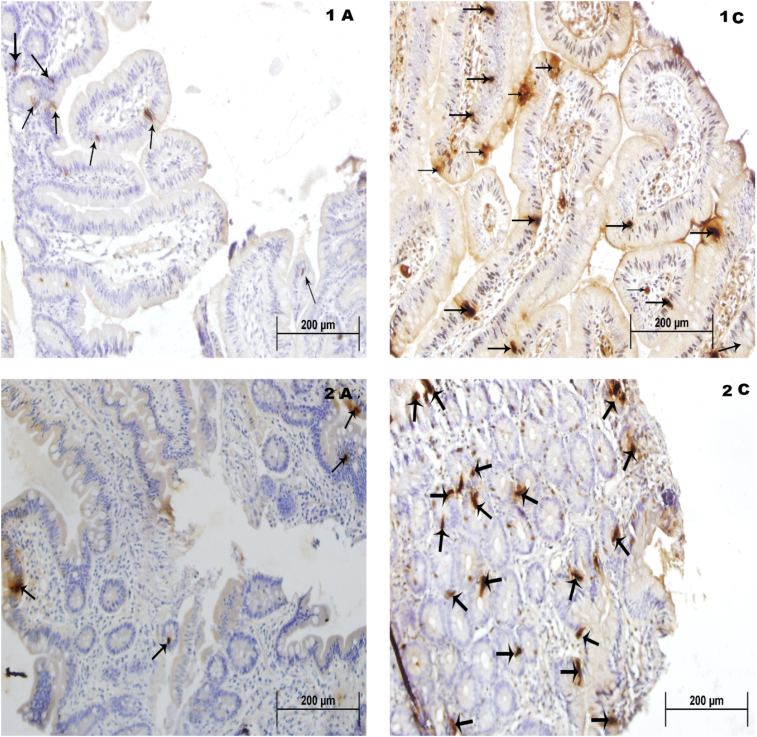
Immunohistochemistry of the intestinal epithelium labeled with monoclonal antibody to GLP-1 showing positive staining in the intestinal L cells indicated by the arrows in patient 1 (1A/left and 1C/right) and patient 2 (2A/left and 2C/right) after RYGB.

The number of active intestinal L cells per field in each region increases in the same patient from point A to point C (Figure 2).

## DISCUSSION

The GIT is an important field for investigation and understanding of the functioning of the epithelial incretin pathway, since it contains L cells and enterohormones, the key mechanisms for glycemic homeostasis, weight loss, nutrient intake, and body's satiety signal^3^. RYGB was described as an anatomical rearrangement that will act as a hormonal trigger in the gastrointestinal system, leading to the induction of intestinal incretin secretion through epithelial sensitization by contact with food intake and absorption in the lumen by the L cell^12^.

The L cell distribution along the GIT and regional expression of the enterohormone varies according to anatomical site. Moreover, immunoreactive L cell activity will also vary in healthy individuals (more proximal) or T2DM patients (more distal)^8^. L cell activity in individuals after bariatric surgery and T2DM remission presented an increased density from the jejunum to the ileum, where significant activity after 80 cm from the jejunum was observed, with intensified activity from 200 cm^10,13,14,15^.

Recent publications and studies demonstrate RYGB influence is also due to the height of the biliopancreatic and food loops, since they exert a great influence on metabolic modulation, nutrient absorption, and epithelial sensitization in the intestinal portion, with greater hormonal secretion and better glycemic control^9^.

It is known that every cell seeks to maintain a low basal activity for its survival, but when stimulated, it starts the metabolic signaling cycle (GLP-1 secretion) incretin by the L cell. In the results of immunoassays at follow-up, an increase in L cell activity is noted in patient 1 (Figure 3) and the same can be observed in patient 2 (Figure 3).

There is a correlation in the metabolic pathway of the stimulation of the distal gastrointestinal epithelium and GLP-1 secretion by L cells^6,7^. RYGB with different length of the intestinal loops could enhance the physiological and biochemical response, aiding to the resumption of intestinal hormonal signaling, leading to a neural response and reduction of appetite and substantial weight loss, as the nutrient stimulating GIT could reach the most distal portion where the largest L cell site is located^11,14^.

Nergaard et al. mentioned that improvement in weight loss occurs with the bioliopancreatic loop with 60 cm and food loops at 150 cm. In our study, patients underwent RYGB with food and biliopancreatic loops standardized at 60 cm, aiming to assess whether there really was a significant difference between the points close to the gastrointestinal anastomosis (GIA= Point A), close to the enteroenteral anastomosis (EEA= Point B= 60 cm distal to the GIA), and 60 cm distal to the EEA (Point C)^15^.

One study investigated how the treatment of severely obese individuals by RYGB helps in weight loss as well as in the control of glycemic homeostasis, an improvement in nutrient induction to the point of contributing for signaling in the lumen of the intestinal mucosa increasing the release of enterohormonal incretins^10^. Rhee et al. found that the anatomical change led to transcriptional modulation altering the secretion of active enteroendocrine L cells after RYGB, that is, the procedure acts as a trigger in the process of response and signaling at the neuroendocrine level^18^.

Another study demonstrates that despite the cell distribution at the L site there is a clear visible immunoactivity at point A (60 cm), the profile of immunomarker cells was maintained at point B, and there is better expression of GLP-1 and signaling increase activity of L cell in point C (120 cm distal to EEA)^1^.

A statistical analysis of the GIT of segments A and C (p=0.001) was performed, considering the significance level of the nonparametric Kruskal-Wallis test established for the value of p<0.001. There was a significant difference between biopsy results in point C (compared to other groups 120 cm from the EEA), with mean cell count of 12.5 (±4.73) and median of 11.5 (8–17).

Incretins secreted by the intestinal L cell also control the level of blood nutrients and thus help in digestion and absorption so that it occurs more slowly, consequently reducing the circulation of nutrient intake^6,7^.

We can conclude that RYGB with food and biliopancreatic loops standardized at 60 cm, it is already possible to observe resumption of L cell signaling and activity in the intestinal epithelium. Through standardization analysis of the points at 60 cm biliopancreatic loop and 120 cm from the alimentary loop, we observed a significant cell density change. This variation may explain the difference in intestinal lumen sensitization and enterohormonal response after RYGB.

## CONCLUSION

The concentration of L cells is higher 60 cm distal to enteroenteral anastomosis when comparing to proximal segments and may explain the difference in intestinal lumen sensitization and enterohormonal response after RYGB.
